# Supplementary material for: Semantic novelty modulates neural responses to visual change across the human brain
Source: Nat Commun. 2023 May 22;14:2910. doi: 10.1038/s41467-023-38576-5 (PMC10203305; doi:10.1038/s41467-023-38576-5)
Supplement: Supplementary file 3 — Reporting Summary [file 41467_2023_38576_MOESM3_ESM.pdf]

nature portfolio

Corresponding author(s): Maximilian Nentwich, Lucas C. Parra

Last updated by author(s): Apr 16, 2023

Reporting Summary

Nature Portfolio wishes to improve the reproducibility of the work that we publish. This form provides structure for consistency and transparency in reporting. For further information on Nature Portfolio policies, see our Editorial Policies and the Editorial Policy Checklist.

Statistics

For all statistical analyses, confirm that the following items are present in the figure legend, table legend, main text, or Methods section.

n/a	Confirmed	
☐	☒	The exact sample size (*n*) for each experimental group/condition, given as a discrete number and unit of measurement
☐	☒	A statement on whether measurements were taken from distinct samples or whether the same sample was measured repeatedly
☐	☒	The statistical test(s) used AND whether they are one- or two-sided<br>*Only common tests should be described solely by name; describe more complex techniques in the Methods section.*
☐	☒	A description of all covariates tested
☐	☒	A description of any assumptions or corrections, such as tests of normality and adjustment for multiple comparisons
☐	☒	A full description of the statistical parameters including central tendency (e.g. means) or other basic estimates (e.g. regression coefficient) AND variation (e.g. standard deviation) or associated estimates of uncertainty (e.g. confidence intervals)
☐	☒	For null hypothesis testing, the test statistic (e.g. *F*, *t*, *r*) with confidence intervals, effect sizes, degrees of freedom and *P* value noted<br>*Give P values as exact values whenever suitable.*
☒	☐	For Bayesian analysis, information on the choice of priors and Markov chain Monte Carlo settings
☒	☐	For hierarchical and complex designs, identification of the appropriate level for tests and full reporting of outcomes
☐	☒	Estimates of effect sizes (e.g. Cohen's *d*, Pearson's *r*), indicating how they were calculated

Our web collection on statistics for biologists contains articles on many of the points above.

Software and code

Policy information about availability of computer code

Data collection	Simuli were presented using PSychtoolbox version PTB_Beta-2014-10-19_V3.0.12 in Matlab 2012b on Windows 7. This requires Gstreamer version 1.10.2. Neural data was recorded from depth and grid electrodes on a neural recording system by Tucker-Davis Technologies (TDT, Alachua, FL, USA). Eyetracking data was recorded with a Tobii TX 300 (Tobii Technology, Stockholm, Sweden). Experiments to detect event boundaries were programed in PsychoPy3, version 2021.1.4 (https://www.psychopy.org/). Custom code was scripted in Matlab to coordinate stimulus presentation through psychtoolbox and neurophysiological and eyetracking data acquisition.
Data analysis	All data analysis was performed in Matlab 2020b and Python 3.10.9. Code for analysis is provided on github (https://doi.org/10.5281/zenodo.7811028) and is built on several packages. The mTRF toolbox version 2.0 (https://github.com/mickcrosse/mTRF-Toolbox/releases/tag/v2.0). Freesurfer version 6 (https://surfer.nmr.mgh.harvard.edu/fswiki/DownloadAndInstall) and the iELVis toolbox (https://github.com/iELVis/iELVis) for electrode location and visualization. iElvis uses Bioimage Suite version 3.01 (https://medicine.yale.edu/bioimaging/suite/lands/downloads/). The CBIG toolbox v.0.8.2 for clustering responses (https://github.com/ThomasYeoLab/CBIG/releases/tag/v0.8.2-Yeo2011_fcMRI_clustering). Labelme version 4.5.6 (https://github.com/wkentaro/labelme/releases/tag/v4.5.6) and Roboflow (Roboflow Inc, Des Moines, Iowa) for face annotation. Face detection was performed using the detectron2 platform v0.5 (https://github.com/facebookresearch/detectron2/releases/tag/v0.5). Saccade novelty was computed with a ResNet-50 trained with SimCLR version 1 (https://github.com/google-research/simclr/releases/tag/1.0). Cortical surface plots are based on field_echos v1 by Gao et al. (30) (https://doi.org/10.5281/zenodo.4362645). Maps for functional and anatomical gradients were obtained with neuromaps v0.0.3 (https://doi.org/10.5281/zenodo.7154329).

For manuscripts utilizing custom algorithms or software that are central to the research but not yet described in published literature, software must be made available to editors and reviewers. We strongly encourage code deposition in a community repository (e.g. GitHub). See the Nature Portfolio guidelines for submitting code & software for further information.

Data

Policy information about availability of data

All manuscripts must include a data availability statement. This statement should provide the following information, where applicable:

- Accession codes, unique identifiers, or web links for publicly available datasets
- A description of any restrictions on data availability
- For clinical datasets or third party data, please ensure that the statement adheres to our policy

The processed results data are available at OSF (https://doi.org/10.17605/osf.io/n6vpc). Source data are provided with this paper.

Human research participants

Policy information about studies involving human research participants and Sex and Gender in Research.

Reporting on sex and gender	The sample size is too small to consider gender segregated analysis. Additionally, the hypothesis does not suggest that there should be a sex and gender difference in basic processing of visual stimuli. Nevertheless, we have collected data from both genders and have reported this in the manuscript.
Population characteristics	Electrophysiology data was obtained from 23 patients with pharmacologically-intractable epilepsy. The mean age is 37.96 years (range 19-58 years) 11 patients are female. Patient demographics are listed in Table S1. The age range and gender distribution of participants in the online experiment is reported in the results section of the paper (N=180, mean age 27.9 years, age range 19-67 years, 60 female).
Recruitment	The human subjects are patients with epilepsy. We minimized the possible effects of this disease on our results by excluding epochs with epileptic activity and electrodes located in the seizure network from analysis and recorded experimental data long after seizures when the electrophysiological signal and the patient's clinical status was back to baseline. Furthermore, our patients have focal epilepsy and only ~18% of the implanted electrodes end up in epileptic tissue (Parvizi, Josef, and Sabine Kastner. "Promises and limitations of human intracranial electroencephalography." Nature neuroscience 21.4 (2018): 474-483)
Ethics oversight	Subjects undergoing invasive electrophysiological recording for clinical purposes were offered to participate in this study and consented prior to data collection. The study was approved by the institutional review board at the Feinstein Institute for Medical Research.

Note that full information on the approval of the study protocol must also be provided in the manuscript.

Field-specific reporting

Please select the one below that is the best fit for your research. If you are not sure, read the appropriate sections before making your selection.

☒ Life sciences ☐ Behavioural & social sciences ☐ Ecological, evolutionary & environmental sciences

For a reference copy of the document with all sections, see nature.com/documents/nr-reporting-summary-flat.pdf

Life sciences study design

All studies must disclose on these points even when the disclosure is negative.

Sample size	Our study is based on recordings from 6328 electrode contacts from 23 patients. This sample size is large compared to previous studies of this kind in this patient population. There is no consensus in the iEEG community regarding statistical procedures, including the determination of sample size: Mercier, Manuel R., et al. "Advances in human intracranial electroencephalography research, guidelines and good practices." NeuroImage (2022): 119438. iEEG studies can be conducted as single or multiple case studies or group studies. The limitation for group studies is sparse sampling. We address this limitation by defining large regions of interests to ensure multiple observations in each area. Our statistical tests include subjects as a fixed effect to account for variation across subjects.
Data exclusions	As described in the methods and as customary in the field electrodes with excessive noise were excluded from analysis. This step is necassary to exlude data with epileptic activity from seizure onset zones. This approach was summarized in: Parvizi, Josef, and Sabine Kastner. "Promises and limitations of human intracranial electroencephalography." Nature neuroscience 21.4 (2018): 474-483.
Replication	The results obtained on cartoons were replicated on documentaries of natural environments of monkeys, comprising one replication of the analysis of film cuts reported in Figure 3 and Figure S10.
Randomization	We used within-subject analysis: All subjects completed the same task. Permutation tests were performed when appropriate to ensure statistical validity of our results.

Blinding	Our study did not include a specific task apart from passive movie watching. Further, we did not conduct any group analysis. Therefore, blinding considerations are not applicable.

Reporting for specific materials, systems and methods

We require information from authors about some types of materials, experimental systems and methods used in many studies. Here, indicate whether each material, system or method listed is relevant to your study. If you are not sure if a list item applies to your research, read the appropriate section before selecting a response.

Materials & experimental systems

n/a	Involved in the study
☒	☐ Antibodies
☒	☐ Eukaryotic cell lines
☒	☐ Palaeontology and archaeology
☒	☐ Animals and other organisms
☒	☐ Clinical data
☒	☐ Dual use research of concern

Methods

n/a	Involved in the study
☒	☐ ChIP-seq
☒	☐ Flow cytometry
☒	☐ MRI-based neuroimaging
